# Intricate disorder in defect fluorite/pyrochlore: a concord of chemistry and crystallography

**DOI:** 10.1038/s41598-017-02787-w

**Published:** 2017-06-16

**Authors:** David Simeone, Gordon James Thorogood, Da Huo, Laurence Luneville, Gianguido Baldinozzi, Vaclav Petricek, Florence Porcher, Joel Ribis, Leo Mazerolles, Ludovic Largeau, Jean Francois Berar, Suzy Surble

**Affiliations:** 1 DEN/Service de Recherches Metallurgiques Appliquees, CEA, Universite Paris-Saclay, F-91191, Centralesupelec/SPMS/UMR-8085/LRC CARMEN, 92292 Chatenay Malabry, France; 2 ANSTO, Lucas Heights, NSW, Australia; 3 Department of Nuclear System Safety Engineering, Nagaoka University of Technology, 1603-1 Kamitomioka, Nagaoka, 940-2188 Japan; 4 LEEL, NIMBE, CEA/CNRS, Universite Paris Saclay, 91191 Gif sur, Yvette France; 5 DEN/Service d’Etude et de Recherches en Mathematiques Appliquees, CEA, Universite Paris-Saclay, F-91191, Centralesupelec/SPMS/UMR-8085/LRC CARMEN, 92292 Chatenay Malabry, France; 6 Institute of Physics ASCR, v.v.i., Na Slovance 2, Prague, Czech Republic; 7 Laboratoire Leon Brillouin, CEA/CNRS Universite Paris Saclay, 91191 Gif sur Yvette, France; 8 Institut de Chimie et des Materiaux CNRS UMR 7182, Universite Paris Est, 2-8 Rue Henri Dunant, F-94407 Vitry Sur Seine, France; 9 C2N/CNRS-Universite Paris-Saclay, Route de Nozay, 91460 Marcoussis, France; 10 Institut Neel, CNRS/UJF UPR 2940, 25 rue des Martyrs BP166, 38042 Grenoble cedex 9, France

## Abstract

Intuitively scientists accept that order can emerge from disorder and a significant amount of effort has been devoted over many years to demonstrate this. In metallic alloys and oxides, disorder at the atomic scale is the result of occupation at equivalent atomic positions by different atoms which leads to the material exhibiting a fully random or modulated scattering pattern. This arrangement has a substantial influence on the material’s properties, for example ionic conductivity. However it is generally accepted that oxides, such as defect fluorite as used for nuclear waste immobilization matrices and fuel cells, are the result of disorder at the atomic scale. To investigate how order at the atomic scale induces disorder at a larger scale length, we have applied different techniques to study the atomic composition of a homogeneous *La*_2_ *Zr*_2_ *O*_7_ pyrochlore, a textbook example of such a structure. Here we demonstrate that a pyrochlore, which is considered to be defect fluorite, is the result of intricate disorder due to a random distribution of fully ordered nano-domains. Our investigation provides new insight into the order disorder transformations in complex materials with regards to domain formation, resulting in a concord of chemistry with crystallography illustrating that order can induce disorder.

## Introduction

**Figure 1:**
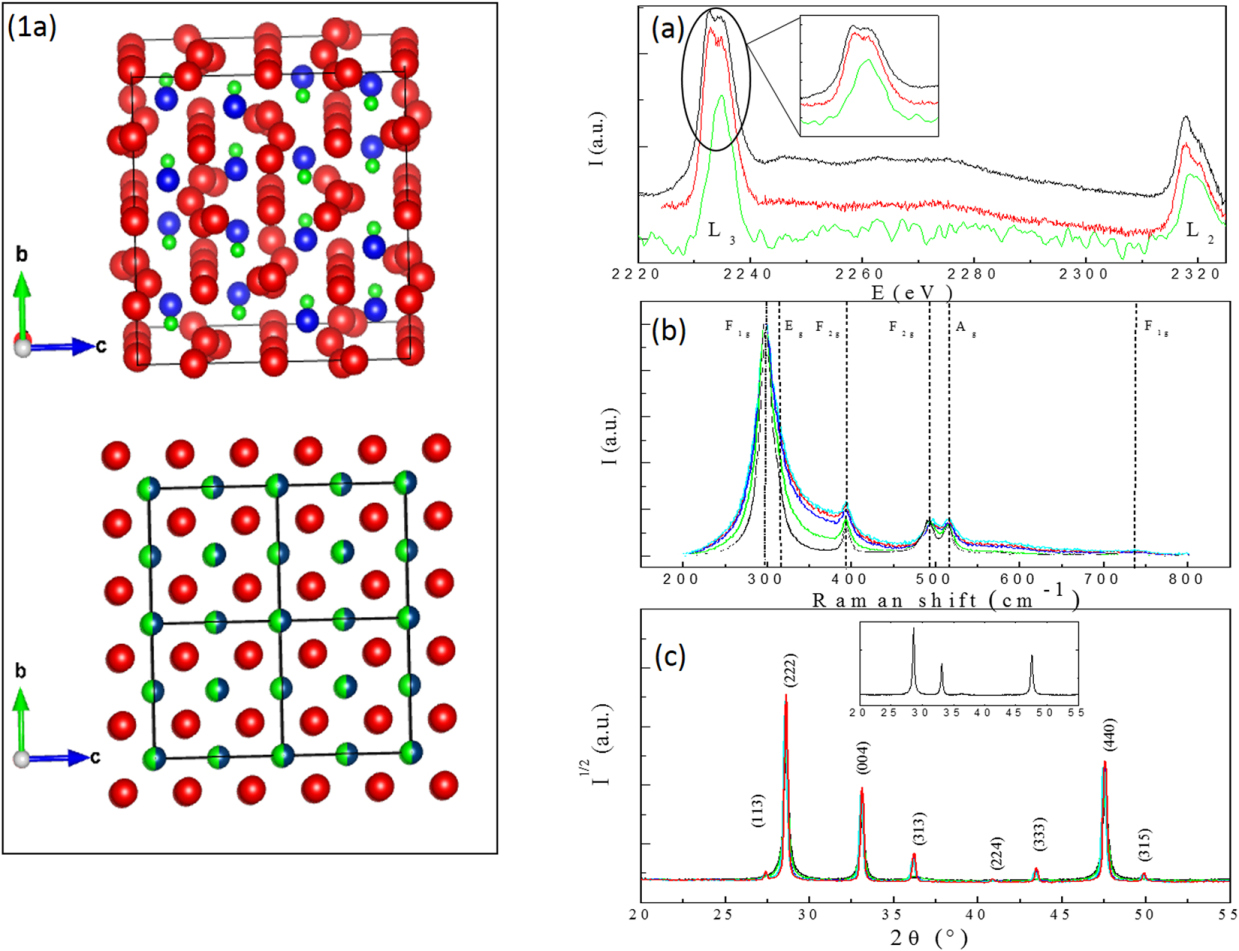
Description of the structures (1a), EELS (**a**), Raman (**b**) spectra and X-ray diffraction patterns (**c**) collected on the *La*_2_ *Zr*_2_ *O*_7_ powders. Figure 1a is a schematic of the pristine (top) and defect fluorite (bottom) pyrochlore structure. Oxygen sites (red) are fully occupied in the pyrochlore structure and ${\frac{7}{8}}^{th}$ occupied in the defect fluorite structure. La (blue) and Zr (green) sites in the pyrochlore structure are equivalent in the defect fluorite decreasing the unit cell by two (black squares). EELS spectra of the micrometric (red, *t* = 350 *nm*), nanometric (black, *t* = 70 *nm*) pyrochlores and cubic *ZrO*_2_ (green: *t* = 250 *nm*) are displayed in Figure a. The Raman spectra (**b**) does not vary with respect to grain size. X-ray diffraction patterns for samples with different grains sizes are plotted in figure c (black: 70 nm, blue: 120 nm, green: 150 nm, cyan: 200 nm, red: 350 nm). The loss of odd X-ray reflexions in samples with small grains is highlighted in the insert.

The properties of a material are intrinsically related to the notion of order and applications that are associated with thermal or ionic conductivities such as barrier coatings or fuel cells are closely related to the ordering of atoms over different length scales^1, 2^. Complex oxides like defect fluorites can be thought of as superstructures of simple atomic building blocks over a length of tens of nanometers, and offer the unique opportunity to tune certain properties^3, 4^ such as ionic conductivity^5, 6^ by modifying these building blocks, i.e. over a length of less than a nanometer. Given that there is a single cation site in defect fluorite, the coordination number is seven for both La and Zr, irrespective of their valence state^7^. Therefore if the samples are transforming from pyrochlore to defect fluorite with respect to grain size a change in local symmetry should also be occurring. To test this assumption, Electron Energy Loss Spectroscopy (EELS) was employed to probe the inner ionisation shell of the *Zr* atom for samples of different grain sizes. Comparison of EELS spectra collected near the *L*_2,3_ edge of *Zr* indicate the local *Zr* symmetry does not vary with respect to grain size as shown in Fig. 1a (two distinct peaks at 2233 and 2235 eV in the *L*_3_ edge are clearly visible for the nanometric (black line) and micro-metric (red line) *La*_2_ *Zr*_2_ *O*_7_ pyrochlore samples. EELS spectrum of cubic *ZrO*_2_, the archetype of the defect fluorite structure, clearly displays a single peak at 2234 eV in the *L*_3_ edge (green line) in agreement with previous studies^8, 9^). These results show that the valence state of *Zr* does not vary in small grain pyrochlores which have been identified via diffraction methods as defect fluorite. To reconcile the charge valence with crystallography, recent diffraction studies have attempted to define this defect fluorite structure as resulting from intricate disorder^5^ associated with a random distribution of Weberite nano domains^6^. This analysis is solely based on the observation of an unusual broadening of the first peaks in an experimental neutron pair distribution function. However, the physical mechanism responsible for the formation of the defect fluorite structure and the stabilization of the claimed Weberite phase at the atomic scale remains unclear.

To gain an insight into this phenomenon, we have engineered nano pyrochlore domains to probe order at different length scales. The pyrochlore we selected was *La*_2_ *Zr*_2_ *O*_7_ because the cation ionic radii ratio is large enough ($\frac{{R}_{La}}{{R}_{Zr}}\mathrm{ > 1}$) to stabilize the ordered pyrochlore structure with the valence states for *La* and *Zr* being 3^+^ and 4^+^ respectively. By decreasing the grain size *t* in this material via a green chemistry method^7^, previous authors^8^ indicated that below a threshold value (100 nanometers), the diffraction pattern is similar to that observed in the defect fluorite. Therefore by combining green chemistry methods to produce homogeneous powders, and the selection of cations of appropriate valences, offers the unique opportunity to understand the defect fluorite/pyrochlore transformation by characterising samples with different grain sizes. Defect fluorite is analogous to the mineral fluorite, with a single cation and anion site (Fig. 1). The random mixing of oxygen and vacancies on a single site increases the number of symmetry operations reducing the pyrochlore unit cell parameter by $\frac{1}{2}$. To achieve the transformation to a defect fluorite, an unusual disordering of cations must simultaneously take place. This order/disorder transition from the pyrochlore to the defect fluorite structure is a very rare example of simultaneous disordering of both anions and cations^9^. Given that there is a single cation site in defect fluorite, the coordination number is seven for both La and Zr, irrespective of their valence state. Therefore if the samples are transforming from pyrochlore to defect fluorite with respect to grain size a change in local symmetry should also be occurring. To test this assumption, Electron Energy Loss Spectroscopy (EELS) was employed to probe the inner ionisation shell of the *Zr* atom for samples of different grain sizes. Comparison of EELS spectra collected near the *L*_2,3_ edge of *Zr* indicate the local *Zr* symmetry does not vary with respect to grain size as shown in Fig. 1a. These results show that the valence state of *Zr* does not vary in small grain pyrochlores which have been identified via diffraction methods as defect fluorite.

**Figure 2:**
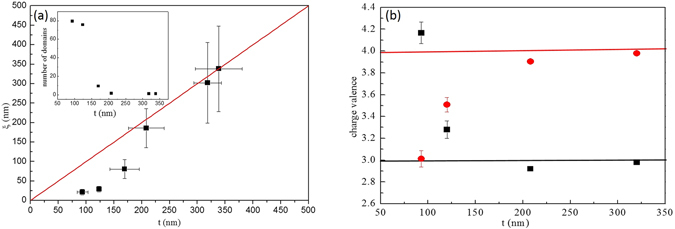
Variation of pyrochlore domains *ξ* (**a**) and valencies of *La* and *Zr* cations (**b**) versus grain size. From the joint refinements of X-ray and neutron diffraction powder patterns, the evolution of La (black squares) and Zr (red dots) valences are plotted versus grain size (**b**) and compared with their nominal values (full lines).

In order to probe symmetries in these samples, Raman scattering was employed (Fig 1b). Regardless of grain size six distinct Raman peaks are always visible. These peaks can be derived via group theory calculations for the pyrochlore structure^10^ (dashed vertical lines on Fig. 1b). The persistence of these peaks disagrees with the appearance of a unique Raman peak as expected for the defect fluorite structure^11^. However, X-ray and neutron diffraction patterns clearly illustrate the loss of odd Bragg peaks which is associated with the pyrochlore/defect fluorite transition versus grain size (Fig. 1c). The characteristic size *t* of diffracting domains derived via analyses of the broadening of even reflexions using Hall Williamson (HW) plots^12^ agrees with the observation of grain sizes performed via Transmission Electron Microscopy (HW curves are plotted in Figure 4 in the supporting information). Surprisingly, the broadening of the odd reflexions is more important as the grain size decreases (Fig. 1c) and these reflexions vanish in samples with the smallest size, thus leading to diffraction patterns similar to the defect fluorite. This analysis suggests the existence of a characteristic length *ξ* extracted from the HW plots of the odd reflexions associated with the coherent diffracting domains smaller than the grain size (Fig. 2a). For small grain sizes *ξ* ≪ t, numerous mesoscopic domains are present in the grain (insert Fig. 2a) whereas few domains are present for *ξ* ≈ *t*, i.e. in samples with large grain sizes. The first bisecting line (red) in Fig. 2a displays the limit between two regimes. From joint neutron and X-ray Rietveld refinements, the valencies of cations were computed via the Bond Valence Model^13^. For samples with large grain sizes, the nominal valencies for *Zr* and *La* are in agreement with the pyrochlore model (Fig. 2b). As the grain size decreases, these valences reach unrealistic values of 2.8 and 4.2 for *Zr* and *La* cations respectively (Fig. 2b). This phenomenon is associated with a largely distorted local environment of the *Zr* cations and is in disagreement with EELS results displayed in Fig. 1a.

To reconcile all of these experimental results, we present a novel approach to explain the observed diffraction patterns. From group theory calculations^14^, defect fluorite can be understood to result from an ensemble average of different perfectly ordered pyrochlore domains of size *ξ* ≪ *t* randomly shifted from the pyrochlore setting by given translation vectors (*Id*|*t*_*j*_) (Table 1 in the supporting information). From this model, the *La* and *Zr* cations are equally distributed at all cationic sites with vacancies spread over all oxygen sites implying a partial ($\frac{7}{8}$) occupation of these sites (Table 1 in the supporting information). The defect fluorite structure is achieved without invoking any simultaneous disordering of anions and cations at the atomic level and it removes the requirement for the appearance of a new phase at the atomic scale. Therefore each domain remains as a pyrochlore of domain size *ξ* in what would be described as defect fluorite via diffraction methods, additionally the EELS and Raman spectra do not vary with grain size as displayed in Fig. 1a and b.

When the number of domains ${(\frac{t}{\xi })}^{3}$ in the grain (*ξ* ≪ *t* are large, the structure factor < *F*_*DF*_(*hkl*) > of the diffraction patterns can be expressed as the product of the pyrochlore structure factor *F*(*hkl*) by an interference function *Z*(*hkl*):

$$ < {F}_{DF}(hkl) > =F(hkl)\sum _{j\mathrm{=1}}^{8}{e}^{i2\pi ({t}_{j}^{h}h+{t}_{j}^{k}k+{t}_{j}^{l}l)}=F(hkl)Z(hkl)$$

where Bragg peaks are labelled by the (*hkl*) triplets, ${t}_{j}^{i}$ is the *i* ^*th*^ component of (*Id*|*t*_*j*_) (the list of these vectors is printed in Table 2 in the supporting information). Summing over all the 8 possible variants (*ξ* ≪ *t*) implies that *Z*(*hkl*) vanishes and the defect fluorite diffraction pattern associated with the null (200), (022), (422), (244) and odd reflexions of the pyrochlore structure is restored (Fig. 1c). By increasing the grain size, the number of pyrochlore domains decreases (insert in Fig. 2a) this results in the *Z*(*hkl*) for these reflexions no longer being reduced and thus the pyrochlore diffraction pattern is re-established.

**Figure 3:**
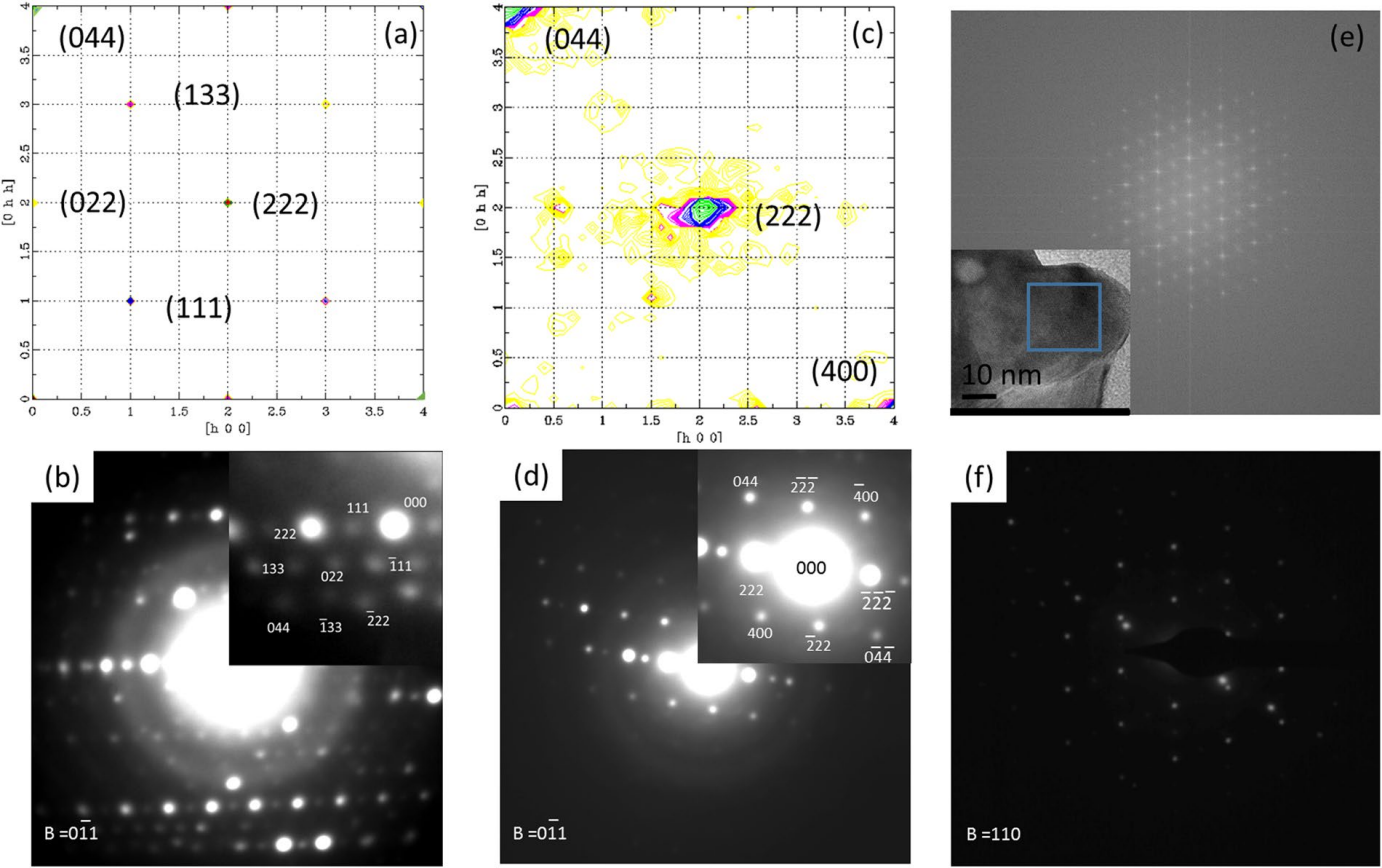
Simulated and experimental High Resolution TEM patterns. Comparison between simulated TEM diffraction for a disorder free pyrochlore (Fig. 3a) and the virtual crystal (Fig. 3c) and experimental data collected on samples with the largest (Fig. 3b) and the smallest (Fig. 3d) grain size. Fourier transformations (Fig. 3e) of a HR TEM picture performed in a volume *ξ* ^3^ (blue square and experimental TEM pictures collected in the volume *t* ^3^ (Fig. 3f)).

To assess our model, simulated TEM diffraction patterns of a disorder free pyrochlore and a large virtual crystal resulting from a random distribution of shifted pyrochlore domains of size *ξ* are plotted on Fig. 3a and c. Simulated TEM diffraction patterns resulting from electron diffraction derived from a disorder free pyrochlore structure (Fig. 3a) and the virtual crystal (Fig. 3c) are in fair agreement with experimental results obtained on samples with the largest (Fig. 3b) and smallest (Fig. 3d) grain size. Figure 3c displays the loss of the odd diffraction spots in agreement with experimental TEM patterns (Fig. 3d). Fourier transformations (Fig. 3e) of a HR TEM picture calculated in the volume *ξ* ^3^ (blue square in the insert) neglecting diffraction display a pyrochlore structure for the samples with the smallest grain size. Moreover, experimental TEM pictures collected in the volume *t* ^3^ in the same sample are similar to TEM diffraction patterns of a defect fluorite structure (Fig. 3f). These experimental results thus ensure that the structure is always pyrochlore in samples with small grain sizes. The vanishing of diffraction spots only results from interference between incident and scattered waves.

Simulated X ray powder patterns of the disorder free and the virtual pyrochlore crystals (supplementary Fig. 4a) display a similar progression as the experimental X-ray diffraction patterns (Fig. 1c) indicating the effect of *Z*(*hkl*). From our model, experimental neutron pair distribution functions are able to provide separate results consistent with the local and long range structures (supplementary Fig. 4b) which were also computed and agree closely with published results^6^. Therefore, no distortion or broadening of the first peaks of *G*(*r*) (supplementary Fig. 6b) can be observed in the virtual crystal ensuring the conservation of the local environment of cations in domains in agreement with EELS measurements.

In summary, a combination of spectroscopic and diffraction techniques has provided a new insight to demonstrate that order can generate disorder in materials. For the first time, we demonstrate that the defect fluorite structure observed in diffraction patterns of *La*_2_ *Zr*_2_ *O*_7_ is only due to the interference of scattering waves between shifted pyrochlore domains. The intricate disorder resulting from a random distribution at the mesoscopic scale of well ordered pyrochlore domains at the atomic scale leads to structures that are apparently disordered. The existence of interfaces resulting from this intricate disorder must then be taken into account to model the transport properties in these materials. The model presented in this work can easily be extended to understand the aging of a large class of complex oxides such as spinels^15^ with practical applications ranging from fuel cell production to nuclear waste form design and management.

## Methods

An aqueous solution of *La*(*NO*_3_)_3_ − 6(*H*_2_ *O*) with a purity of 99.999% and *ZrOCL*_2.8_(*H*_2_ *O*) with a purity of 99.5% (Aldrich products) were combined. Oxalic acid (Normapur, Prolabo) was slowly added to the mixture in order to induce the precipitation. The final product was produced by the calcination of the precipitate at different temperatures ranging from 1073 K to 1773 K for three hours under atmospheric conditions resulting in homogeneous *La*_2_ *Zr*_2_ *O*_7_ samples of different grain sizes.

Raman spectra were collected on a Renishaw spectrometer equipped with a Notch filter. The 532 nm excitation laser lines were used in order to avoid any luminescence signal. Four strong peaks are visible in the Raman spectra with another visible in the shoulder of the strongest peak (340 cm^−1^) and a small peak at 740 cm^−1^ (small bump in Fig. 1b). These results are in agreement with group theory calculations of a perfect pyrochlore structure (peaks are labelled by their irreducible representations) and previously published Raman spectra^10^. To check the quality of samples, a zoom of the Raman spectra collected on the different samples is plotted in Figure 5 in the supplementary information. No peaks associated with *CaCO*_3_ (intense peaks at 1354 *cm* ^−1^ and 1603 *cm* ^−1^) can be observed in this zoom assessing that no carbonate is present in the pyrochlore samples in agreement with x ray diffraction patterns.

EELS spectra were collected on a FEI Tecnai Scanning Transmission Electron Microscope operating at 200 kV with a GATAN Gif electron spectrometer. The EELS collection angle was 16 milli radiant and spectra were collected near the *L*_2,3_ edge for zirconium. All spectra are collected with an energy resolution of 0.05 eV. EELS spectra collected on micrometric (black line), nanometric *La*_2_ *Zr*_2_ *O*_7_ (red line) and cubic *ZrO*_2_ stabilized by a 8% atomic fraction of Yttrium (green line), the archetype of defect fluorite structures, display distinct shapes near the *L*_3_ edge. Pyrochlore samples exhibit two distinct peaks separated by 2 eV whereas only a single peak can be observed in the cubic-*ZrO*_2_ sample at the *L*_3_ edge (zoom in Fig. 1b) in agreement with previous studies^4^. The existence of these two distinct peaks observe in the nanometric and the micrometric samples points out that the Zr coordination in nanometric *La*_2_ *Zr*_2_ *O*_7_ samples is similar to the Zr coordination in a perfect pyrochlore structure. These two peaks are associated with dipole-allowed transitions and correspond to the *T*_2*g*_ and *E*_*g*_ electronic states^16, 17^, and do not exist in cubic *ZrO*_2_, the archetype of a defective fluorite structure (green curve in Fig. 1b).

X-ray diffraction patterns were collected in Bragg-Brentano geometry using a Bruker D8 Discover diffractometer. The instrument was equipped with a parabolic *Göbel* mirror and a line focus *CuKα* radiation source. The nearly parallel incident X-ray beam was collimated with 0.05 × 6 mm primary slits. Radial sollers were used to decrease the intensity of the background and the diffraction patterns were collected on a position-sensitive detector (Vantec detector).

Neutron diffraction patterns were collected on the high resolution 3T-2 diffractometer at the Laboratoire Leon Brilloiun at Saclay (France) in transmission mode. The wavelength of the neutron beam was 0.123 nm.

Joint Neutron and X-ray Rietveld refinements were performed using Jana^18^ and Xnd^19^ software to extract information on the structure and the micro structure of samples versus grain sizes. For all Rietveld refinements, reliability factors *R*_*wp*_ and *R*_*Bragg*_ were smaller than 10% and 5% insuring the quality of refinements. To study in detail the micro structure of samples, Le bail fits of the X-ray diffraction patterns were performed. The instrumental broadening was altered during the refinement of the diffraction data obtained for the samples annealed at 1773K, i.e. the value of the largest grain size was found to be in agreement with the instrumental parameters. For the remaining refinements, this instrumental broadening was fixed and only the broadening due to the sample, *β* was refined. These refined sample broadening values *β*cos(*θ*) were then plotted versus sin(*θ*) in the HW plots (Fig. 6 in the supporting information). HW plots allow for separate strain (slopes) and size (intercept at the origin) broadening. Moreover, the grain sizes tg extracted from these HW plots are in good agreement with TEM observations thus adding to the validity of our analysis. No variation of unit cell parameters was observed and the maximum value of the strain fields extracted from the slopes of the HW plots^12^ of all reflexions are similar. This indicates that the defect fluorite/pyrochlore transition is not driven by the elastic fields in agreement with non ferroic phase transitions^20^. Joint neutron and X-ray patterns were refined using the defect fluorite model allowing the extraction of both accurate partial occupation of the cation and oxygen atomic positions. From this analysis it is possible to extract the characteristic Zr-O/La-O lengths and calculate the average charge of cations via the Bond Valence model as displayed in Fig. 2b. The purpose of this figure is to demonstrate that such charge valences for the *La* and *Zr* cations are unrealistic in the context of a defect fluorite structure.

Selected area TEM diffraction patterns were acquired on *La*_2_ *Zr*_2_ *O*_7_ powders deposited on a carbon support film grid using a JEOL 2100 transmission electron microscope operating at 200 keV. The High Resolution TEM pictures were collected on powders deposited on a copper grid using a 200 keV TITAN microscope and diffraction patterns were collected with a 70 nm aperture.

The simultaneous condensation of four L points of the Brillioun zone of the $Fm\bar{3}m$ space group describing the defect fluorite structure (Fig. 1) leads to a non standard crystallographic group associated with 8 × 192 = 1536 symmetry operations. This space group is the direct product of the $Fd\bar{3}m$ space group by a coset of 8 pure translation operations (*Id*|*t*_*j*_) printed in Table 1 in the supporting information. The action of each (*Id*|*t*_*j*_) on the Wyckoff positions associated with vacancy (8a), *Zr* (16c) and *La* (16d) cations on pure pyrochlore ($Fd\bar{3}m$ space group) is listed in Table 2 in the supporting information.

The extinction rules for the defect fluorite structure are given by:

1 $$ < {F}_{DF}(hkl) > =F(hkl)Z(hkl)=F(hkl\mathrm{)(1}+{e}^{i\pi (h+k+l)}\mathrm{)(1}+{e}^{i\frac{\pi }{2}(h+k)}+{e}^{i\frac{\pi }{2}i(k+l)}+{e}^{i\frac{\pi }{2}(h+l)})$$

The structure factor is null for the (200), (022), (422), (244) and odd reflexions. All reflexions of the $Fm\bar{3}m$ space group associated with defect fluorite are restored assuming ${a}_{DF}=\frac{{a}_{pyrochlore}}{2}$.

All diffraction simulations were performed using the DISCUS package^21^. Pyrochlore unit cells were expanded along all directions to model a 80 × 80 × 80 *nm* ^3^ crystal. Eight distinct shifted domains were randomly created to generate a virtual crystal. A positive correlation between cells of the same nature were chosen to reproduce the characteristic size of mesoscopic domains *ξ* measured by X-ray diffraction (the probability of having different unit cells of same domains is equal to 20%). Because no variation of the unit cell parameters were observed in experimental X-ray patterns, no distortion of the unit cells were introduced in simulations. TEM diffraction patterns were then computed for the virtual and perfect crystals within the kinematic approximation assuming an infinite coherent length. Broadening of the intensity of the (222) reflexion observed on the virtual crystal is due to the finite size of domains. Simulated X-ray powder diagrams (Fig. 4a in the supporting information) were calculated using the resolution function of the D8 discover allowing for a direct comparison of simulated patterns with the experimental ones (Fig. 1c). High frequency oscillations observed in the background of the powder diagrams are due to the finite size of simulation boxes (the coherent length is always infinite in the DISCUS simulations). Moreover, simulated neutron pair distribution function *G*(*r*) for the perfect and virtual crystals were also computed (supplementary Fig. 4b) using realistic resolution functions from the Nanoscale Ordered materials and Diffraction at the Spallation Neutron Source beam line at Oak Ridge National Laboratory (*Q*_*max*_ = 3.1 *nm* ^−1^). These calculations allow a direct comparison of our simulations with published experimental data and does not take into account the impact of nano domains on the distortion and the broadening of first peaks of *G*(*r*) as claimed for other pyrochlores^6, 22^.

## Electronic supplementary material

supplementary information
