# Supplementary material for: Intricate disorder in defect fluorite/pyrochlore: a concord of chemistry and crystallography
Source: Sci Rep. 2017 Jun 16;7:3727. doi: 10.1038/s41598-017-02787-w (PMC5473899; doi:10.1038/s41598-017-02787-w)
Supplement: Supplementary file 1 — supplementary information [file 41598_2017_2787_MOESM1_ESM.pdf]

Intricate disorder in defect fluorite/pyrochlore: a concord of chemistry and crystallography

David Simeone[1], Gordon James Thorogood[2], Da Huo[3], Laurence Luneville[4], Gianguido Baldinozzi[1], Vaclav Petricek[5], Florence Porcher[6], Joel Ribis[1], Leo Mazerolles[7], Ludovic Largeau[8], Jean Francois Berar[9], Suzy Surble$[3]

[1] DEN/Service de Recherches Metallurgiques Appliquees, CEA, Universite Paris-Saclay, F-91191, Centralesupelec/SPMS/UMR-8085/LRC CARMEN, 92292 Chatenay Malabry, France

[2]ANSTO, Lucas Heights, NSW, Australia and Department of Nuclear System Safety Engineering, Nagaoka University of Technology, 1603-1 Kamitomioka,Nagaoka 940-2188, Japan

[3]LEEL, NIMBE, CEA/CNRS, Universite Paris Saclay, 91191 Gif sur Yvette, France

[4]DEN/Service d'Etude et de Recherches en Mathematiques Appliquees, CEA, Universite Paris-Saclay, F-91191, Centralesupelec/SPMS/UMR-8085/LRC CARMEN, 92292 Chatenay Malabry, France

[5]Institute of Physics ASCR, v.v.i., Na Slovance 2, Prague, Czech Republic

[6]Laboratoire Leon Brillouin, CEA/CNRS Universite Paris Saclay, 91191 Gif sur Yvette, France

[7]Institut de Chimie et des Materiaux CNRS UMR 7182, Universite Paris Est, 2-8 Rue Henri Dunant, F-94407 Vitry Sur Seine, France

[8]C2N/CNRS-Universite Paris-Saclay, Route de Nozay, 91460 Marcoussis, France

[9]Institut Neel, CNRS/UJF UPR 2940, 25 rue des Martyrs BP166, 38042 Grenoble cedex 9, France

As supporting information, the table of the 8 different translations vectors (I_d|t_j) forming the coset G is given, printed in fractional coordinates of the perfect pyrochlore structure (table 1).

(Id|t_1)	(Id|t_2)	(Id|t_3)	(Id|t_4)	(Id|t_5)	(Id|t_6)	(Id|t_7)	(Id|t_8)	average
8a	8a	48f	48f	48f	48f	48f	48f	1/8
16c	16c	16d	16c	16d	16d	16c	16c	½
16d	16d	16c	16d	16c	16c	16d	16d	½

Table 1: Action of (I_d|t_j) vectors on cations and vacancy Wyckoff sites in the pyrochlore structure. The occupancies of vacancies, Zr and La cations result from a simple averaging of Wyckoff positions over 8 domains (last column).

Cations occupy 16d and 16c sites with the same probability, leading to the same occupation by Zr and La atoms of 4a Wyckoff positions in the defect fluorite. Vacancies are mixed with oxygen atoms (8b and 48f sites), leading to a partial occupation equal to 7/8 of oxygen's sites of 8c Wyckoff positions in the defect fluorite.

The list of different translations vectors (I_d|t_j) of the coset G is printed in table 2. These vector define eight different domains.

(Id|t_1)	(Id|t_2)	(Id|t_3)	(Id|t_4)	(Id|t_5)	(Id|t_6)	(Id|t_7)	(Id|t_8)
0.00	0.50	0.25	0.00	0.25	0.50	0.75	0.75
0.00	0.50	0.25	0.25	0.00	0.75	0.50	0.75
0.00	0.50	0.00	0.25	0.25	0.75	0.75	0.50

Table 2: Eight different translations vectors (I_d|t_j) of the coset G. These vectors are printed in fractional coordinates of the perfect pyrochlore structure

Figure 4a displays simulated X ray powder patterns of the pyrochlore structure and the virtual crystal associated with different variants. These diagrams plotted in a square root scale (a) exhibit a fair agreement with experimental ones displayed in Fig 1c. The long range order interference between different variants leads to the vanishing of odd reflexions. Small oscillations of the background are induced by the finite size of the simulation boxes. Total neutron pair distribution functions for the perfect and the virtual crystals convoluted with realistic broadening functions are plotted in Figure 4b. Simulated neutron pair distribution functions are calculated for the pyrochlore structure (black line) and the virtual crystal (red line). No distortion nor peaks broadening can be observed. This insures that the short range order is similar in the pyrochlore structure and the virtual crystal, in agreement with EELS measurements.

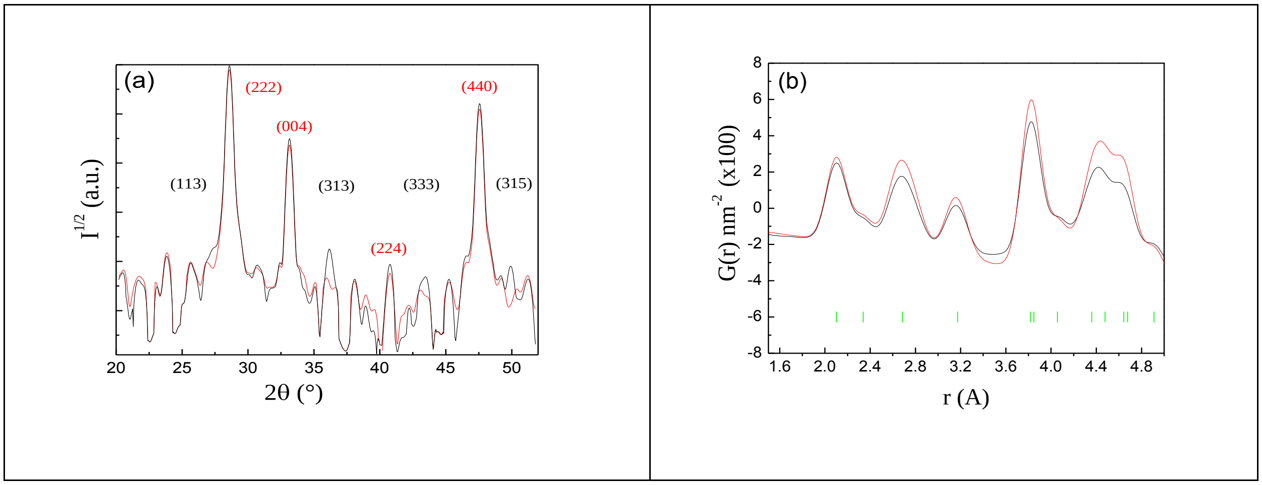

Figure 4: **Simulated X-ray powder diagrams (a) and total neutron pair distribution functions G(r) for the pristine pyrochlore and the virtual crystal (b).** Even reflexions are unchanged and odd reflexions vanish for the virtual pyrochlore (black : pyrochlore, red : virtual crystal). Similar peaks (below 0.5 nm) can be observed in G(r) of the pyrochlore (black line) and the virtual crystal (red line) (distances extracted from Rietveld refinements: green bars).

To study the quality of La_2Zr_2O7 samples studied, a zoom of the Raman spectra for Raman shift varying from 1300 cm^{-1} to 1700 cm^{-1} is presented in Figure 5. No peaks associated with $CaCO_3$ (intense peaks near 1354 cm^{-1} and 1603 cm^{-1}) can be observed in this zoom, in agreement with X ray diagrams assessing the quality of studied samples.

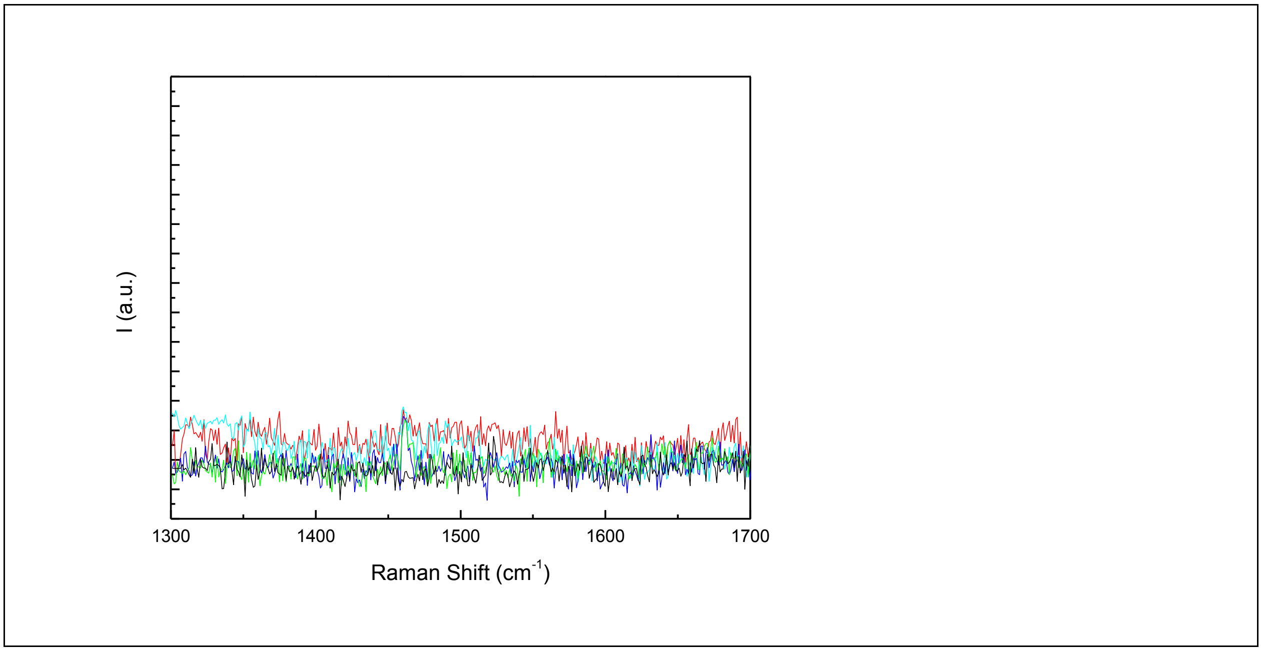

Figure 5: **Zoom of Raman scattering spectra collected for all studied samples**. No Raman peaks can be observed in this range (black: 70 nm, blue: 120 nm, green: 150 nm, cyan: 200nm, red: 350 nm)

Hall Williamson plots extracted from X ray diffraction patterns are displayed in Figure 6 (β is the integral breath associated with the Bragg angle θ). The evolution of the integral breadth β corrected from instrumental broadenings of Bragg reflexions allows to estimate the grain size (intercept at the origin) and the micro strain (slope). The grain size t is extracted from the plots of even reflections and the ordered domain size ξ results from the analysis of plots associated with odd reflections. Grain sizes extracted from HW plots agree with direct TEM observations. Slopes of different HW curves evolve neither with the grain size nor with the evenness of Bragg reflections. This point assesses that defect fluorite/pyrochlore transformation is not associated with a local strain field as expected for a non ferroic transition[25].

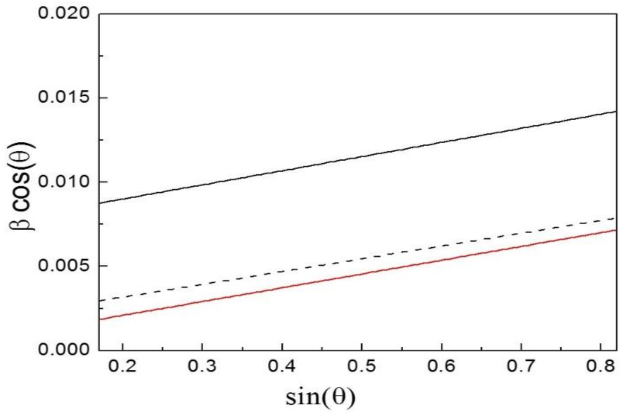

Figure 6: **HW plots of even reflexions and odd reflexions extracted from Bragg reflexions collected on X-ray diagrams** (black full line: 70 nm, odd reflexions; black dashed line: 70 nm, even reflexions; red line: 350 nm, even and odd reflexions.
